# Congenital urethral dilatation in cattle calves: Diagnosis and surgical intervention

**DOI:** 10.14202/vetworld.2020.261-265

**Published:** 2020-02-11

**Authors:** Magda Mahmoud Ali, Kamal Hany Hussein, Ahmed Sadek, Abdelbaset Eweda Abdelbaset

**Affiliations:** 1 Department of Surgery, Anesthesiology and Radiology, Faculty of Veterinary Medicine, Assiut University, Assiut 71526, Egypt; 2 Department of Surgery, College of Veterinary Medicine, Kangwon National University, Chuncheon, Gangwon 24341, South Korea; 3 Department of Animal Medicine, Clinical Laboratory Diagnosis, Faculty of Veterinary Medicine, Assiut University, Assiut 71526, Egypt; 4 National Research Center for Protozoan Diseases, Obihiro University of Agriculture and Veterinary Medicine, 2-13 Inada-cho, Obihiro, Hokkaido 080-8555, Japan

**Keywords:** congenital anomalies, urethral dilatation, urethrostomy

## Abstract

**Background and Aim:**

Congenital anomalies of the urinary system are common affections in ruminants. Dilatation of the pelvic urethra is one of these affections in which the pelvic urethra dilated than normal diameter. This study aimed to explain the diagnosis and surgical treatment of urethral dilatation in cattle calves.

**Materials and Methods:**

Twenty-three bull calves (2-7 months old) were presented with a history of stranguria, tenesmus, and straining. Diagnosis of urethral dilatation was relied on the case history and clinical examination and was confirmed using survey and contrast radiography, ultrasonography, and biochemical tests. Treatment was done by urethrostomy under the effect of local infiltration analgesia.

**Results:**

Physical examination revealed the presence of an oval, firm, and painless swelling at the perineal region, starting just below the anus and extended to the base of the scrotum. The owners reported that the initial swelling size and severity of symptoms increased with the progress of animal age. Biochemical findings revealed non-significant changes in blood urea nitrogen and creatinine levels. Radiographic findings showed an oval radiopaque mass. However, a well-demarcated structure with acoustic enhancement was detected on ultrasonographic examination. Urethrostomy resulted in a successful outcome of all cases.

**Conclusion:**

Depending on these findings, ultrasonography is the most reliable diagnostic tool and urethrostomy is the intervention of choice with acceptable results for diagnosis and treatment of urethral dilatation in cattle calves, respectively.

## Introduction

Urogenital congenital defects were reported in ruminants with variations in the frequency of occurrence [1-3]. The abnormalities of the urinary system are more common in small ruminants than in large ruminants and more frequently in goats than in sheep [4]. The male urethra consists of pelvic and extrapelvic parts, connecting the urinary bladder to the penis [2].

Congenital anomalies involving the urethra of cattle have been described including dilatation, hypospadia, epispadia, ectopic urethra, imperforate urethra, and deformities of the urethral external [5-11]. Congenital urethral dilatation was reported in kids [12-15] and bull calves [8,16-18]. However, still, there are limited reports about the urethral dilatation diagnosis techniques, although the rate of urethral dilatation incidence started to increase.

This study aimed to explain the diagnosis and surgical treatment of urethral dilatation in cattle calves.

## Materials and Methods

### Ethical approval and Informed consent

All animal procedures were conducted in compliance with the guidelines approved by our Institutional Animal Care and Use Committee (Assiut University, Egypt). All procedures were performed with the owners’ consent.

### Animals, case history, and clinical examination

This study was conducted on 23 cattle calves aged from 2 to 7 months, admitted to the Veterinary Teaching Hospital, Faculty of Veterinary Medicine, Assiut University, Egypt, from March 2010 to March 2019. Calves had a history of progressive development of swelling in the perineal region that appeared a few days after birth accompanied by stranguria, tenesmus, dribbling of urine, and straining. All animals underwent a thorough clinical examination according to Radostits *et al*. [7]. General condition of the animal, rectal temperature, heart rate, and respiratory rate as well as shape, size, and consistency of these swellings was examined.

### Radiographic and ultrasonographic examination

Survey radiography was applied to the perineal region in latero-lateral projection. Positive-contrast urethrography was performed under the effect of xylazine HCl 2% (Xyla-Ject^®^, ADWIA Co., Egypt) in a dose of 0.1 mg/kg body weight and local application of Xylocaine HCl 2% jelly (AstraZeneca Co., Egypt) on a French Foley urinary catheter. After insertion of the catheter into the distal urethra to the level of the proximal sigmoid flexure, the urethral lumen was compressed manually at the base of the catheter. The urine in the swelling was aspirated and replaced with 120 ml of sodium iothalamate (Conray 400, Mallinckrodt Inc., St. Louis, Missouri, USA) diluted to a concentration of 200 mg iodine/ml.

Ultrasonographic examination of the swellings using 8-10 MHz linear transducer (Pie medical Aquila Pro Vet, Maastricht, Holland) was performed for 14 cases in the standing position and without the use of anesthetics.

### Biochemical analysis

Whole blood samples were collected from the jugular vein of calves in 5 mL plain Vacutainer tubes. The samples were centrifuged, for 15 min at 1500 g speed, and sera were then harvested and preserved at −20°C until used. Serum levels of creatinine and blood urea nitrogen (BUN) were measured through commercial test kits (Spinreact, Girona, Spain), using ultraviolet (UV) spectrophotometer (Optizen 3220 UV, Mecasys Co. Ltd., Korea).

### Surgical intervention

The perineal region was prepared for aseptic surgery by clipping, shaving, and scrubbing with betadine solution. After local infiltration analgesia using lidocaine HCl 1% (Debocaine^®^, El-Nasr Pharm Chemicals Co., Egypt), an elliptical skin excision was performed at the lower part of the swelling. The subcutaneous tissue was dissected followed by incision of the urethra. After evacuation of the swelling contents and flushing with normal saline 0.9%, a Foley catheter was inserted into the urethra and was directed toward the urinary bladder to ensure the patency of the urethral lumen and absence of other causes of urethral obstruction such as urethral calculi. A permanent fistula was created by suturing of the urethral mucosa to the skin using non-absorbable silk suture material No.1 (Silk^®^, USP, England) in a simple interrupted pattern.

### Post-operative care

A mixture of penicillin G and streptomycin (Vetrocin^®^, El-Nasr Pharm Chemicals, Egypt) was administered intramuscularly every 12 h for a period of 5 days. Follow-up of all cases was done for 3 months.

### Statistical analysis

Data of biochemical analysis are expressed as mean ± standard deviation. The statistical analysis was performed using the SPSS 19.0 software (Chicago, IL, USA). Student’s t-test was used to identify significant differences.

## Results

### Clinical findings

**Figure-1:**
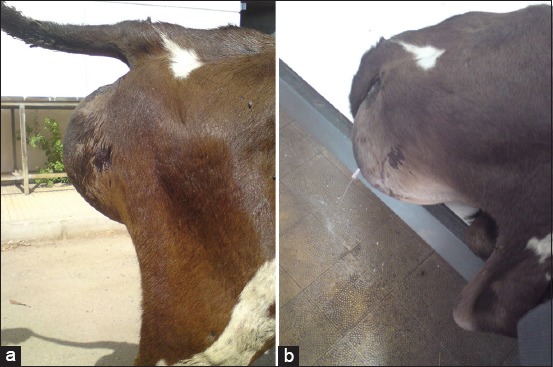
(a) The urethral dilatation appears as an oval swelling in the perineal region. (b) Exploratory puncture of the swelling yielded a clear uriniferous fluid in 12 cases.

**Figure-2:**
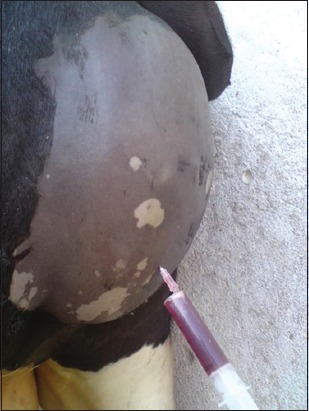
Aspiration of the swelling content revealed the presence of clotted blood in one case.

Clinical examination revealed normal rectal temperature, pulse rate, and respiratory rate in all cases except in two calves that were feverish with abnormal pulse and respiratory rates. The swelling was fluctuant, painless, and extended on the perineal region, from just below the anus till the base of scrotum (Figure-1a). The size and consistency (tensed or relaxed) varied according to the filing status of the swelling. Bulging of the swelling and pulsation of the proximal urethra were observed in all cases during urination. Urine dripped passively from the penis for several minutes after the urethral pulsation had ceased. Direct pressure on the swelling resulted in a further release of urine. Rectal examination was possible in two cases and revealed moderate-to-severe distension of the urinary bladder. Exploratory puncture of the swelling was done under complete aseptic conditions and yielded a clear uriniferous fluid in all cases (Figure-1b). In two cases, the aspirate was turbid and mixed with pus while it was mixed with clotted blood in one case (Figure-2).

### Biochemical findings

**Figure-3:**
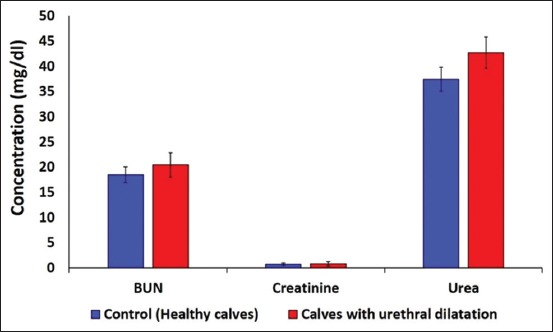
Biochemical analysis of blood urea nitrogen, creatinine, and urea in calves suffered from urethral dilatation compared to healthy calves.

BUN and creatinine levels were not significantly different in calves with urethral dilatation compared to healthy calves. The measured values are recorded in Figure-3.

### Radiographic and ultrasonographic findings

**Figure-4:**
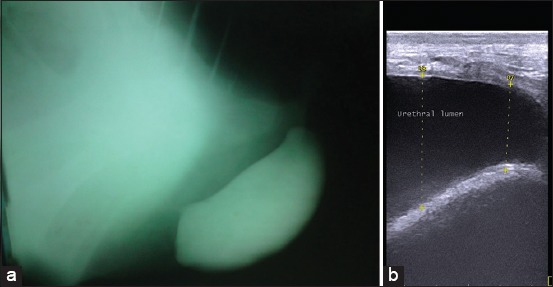
(a) Positive-contrast urethrogram shows a radiopaque oval mass which can easily define from surrounding soft tissues. (b) Ultrasonographic examination revealed the presence of an anechoic to hypoechoic homogenous structure with well-demarcated wall.

In positive-contrast urethrogram, a radiopaque oval mass was observed just under the anus (Figure-4a). Ultrasonographic examination revealed the presence of an anechoic to hypoechoic homogenous structure with a well-demarcated wall and acoustic enhancement (Figure-4b). The connection between the diverticulum and the urinary bladder appeared as a narrow anechoic area with a demarcated wall. Variation in the echogenicity with the appearance of hyperechoic crystals was usually attributed to the variation in the concentration of the urine and concurrent diseases of the urinary tract as in cystitis. Thickening of the urethral wall was evident in all cases.

**Figure-5:**
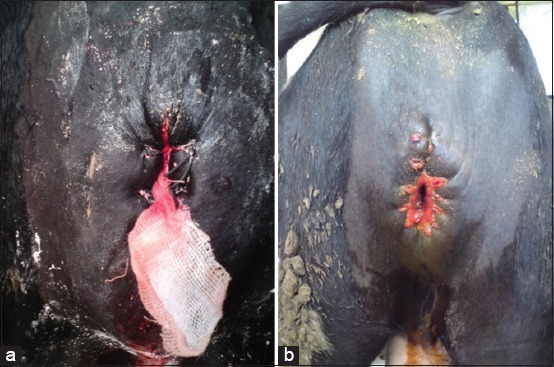
Surgical treatment by urethrostomy resulted in a successful outcome in 21 cases.

**Figure-6:**
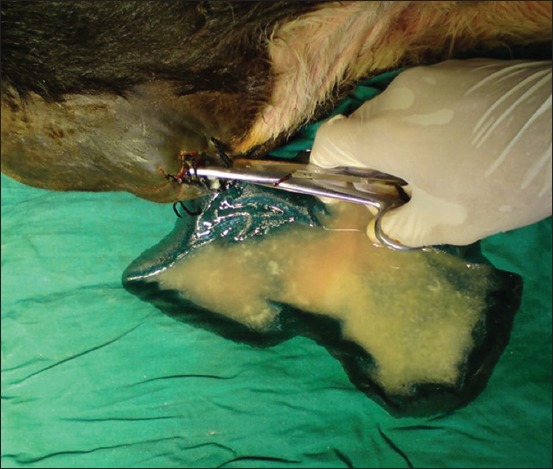
Complication after performing the urethral fistula. The wound closed due to granulation tissue with pus formation; therefore, the swelling occurred again and reoperation was required.

Surgical treatment by urethrostomy resulted in a successful outcome in 21 cases (Figure-5). In two cases, the wound of the urethral fistula closed due to granulation tissue and pus formation; therefore, the swelling occurred again and reoperation of the urethral lumen was required (Figure-6).

## Discussion

Urethral dilatation is one of the urinary tract problems that may be either acquired due to obstruction of the urethra or congenital due to hereditary factors [19-22]. Bacterial urethritis and surgical manipulation could also result in partial obstruction of a part of the urethra which predisposes to urethral dilatation in other parts [12,17]. In our study, the main cause of urethral dilatation was congenital as it was observed a few days after birth. The previous studies have reported that urethral dilatation occurs sporadically and is recognized easily, but sometimes it is neither so easily repaired nor the outcome of its surgical correction has been fully discussed [5,12,16,19,20,23,24]. In our study, the affection was easily recognized from the owner’s history that was appearance of an oval mass in the perineal region interfering with urination few days after birth. However, the urethral swelling should be differentiated from other conditions of the urethra including hematoma or abscess, congenital anomaly of urinary tract, and pathological urethral dilatation [17,25]. In the present work, differential diagnosis was performed by exploratory puncture, biochemical analysis, survey and contrast radiography, and ultrasonography. Sindak *et al*. [25] confirmed the diagnosis of urethral dilatation by exploratory puncture of the swelling, while positive-contrast urethrogram was used by Anderson *et al*. [16] to exclude the rupture of the urethra at the level of the ischial arch and a congenital anomaly of the urinary tract. Ultrasound provided more information needed for differential diagnosis of urethral dilatation compared to other techniques used in our study. The variation in echogenicity of the swelling contents facilitated the exclusion of other affections. Ultrasound was non-invasive and did not require the use of anesthesia or special preparation for the patient [26]. It could be used in standing or recumbent position and could be easily used in field conditions. Contrary to radiography, ultrasound does not involve the use of ionizing radiation. Therefore, it is safe for patients and operators during investigation [27,28]. In addition, Braun mentioned that it is rarely necessary to anesthetize animals for ultrasonography [27].

BUN and creatinine can be used as an index of azotemia due to its simplicity in assessment [7]. In this study, the authors found that BUN and creatinine levels were not significantly different before and after the surgery. This may be attributed to the fact that congenital urethral dilatation did not interfere with urine flow and may be associated only with urine accumulation at the dilated part of the urethra. Animals with urethral dilatation are usually suffered from bacterial urethritis and/or cystitis as a result of accumulation of urine in the swelling for a long period which facilitates the ascending infection [16,29]. In addition, rupture of the urinary bladder may ensue if the condition was associated with aplasia of the penis [30-33]. Due to these complications, surgical intervention in these cases is required [12,32]. In the current study, surgical treatment by urethrostomy resulted in a successful outcome in 21 cases while stricture, reobstruction, and presence of little amount of pus were recorded in two cases 3 weeks post-operative.

## Conclusion

Urethral dilatation is one of the urogenital anomalies affecting cattle calves. Differential diagnosis for such condition is the best done with ultrasonography compared to other diagnostic techniques as radiography. Surgical treatment by performing a permanent urethrostomy was shown to be a successful technique, which helps to save animal life and to improve the animal’s marketability.

## Authors’ Contributions

MMA contributed to the conception, design, provision of field sample, and drafting of the manuscript. AS and AEA contributed to the provision of field samples and drafting the manuscript. KHH contributed to the conception, design, provision of field sample, data analysis and interpretation, and wrote and approved the final draft of the manuscript. All authors contributed to the final editing and approval of the manuscript.
